# Neurological and neuropsychiatric manifestations of post-COVID-19 condition in South America: a systematic review of the literature

**DOI:** 10.1055/s-0044-1779504

**Published:** 2024-02-05

**Authors:** Luisa Pereira de Oliveira Zanetti Gomes, Camila Marinelli Martins, Elis Carolina Pacheco, Letícia Simeoni Avais, Pollyanna Kássia de Oliveira Borges

**Affiliations:** 1 Universidade de Joinville, Departamento de Medicina, Joinville SC, Brazil.; 2 Universidade Estadual de Ponta Grossa, Departamento de Medicina, Ponta Grossa PR, Brazil.; 3 Universidade Estadual de Ponta Grossa, Departamento de Odontologia, Ponta Grossa PR, Brazil.; 4 Universidade Estadual de Ponta Grossa, Departamento de Saúde Pública, Ponta Grossa PR, Brazil.

**Keywords:** Post-Acute COVID-19 Syndrome, Neurologic Manifestations, Mental Health, Síndrome Pós-COVID-19 Aguda, Manifestações Neurológicas, Saúde Mental

## Abstract

**Background** The post-COVID-19 condition is a major modern challenge in medicine and has a high global impact on the health of the population.

**Objective** To determine the main neurological and neuropsychiatric manifestations after acute COVID-19 infection in South American countries.

**Methods** This is a systematic review study, registered on the PROSPERO platform following the PRISMA model. 4131 articles were found with the search strategies used. Neurological and neuropsychiatric manifestations were investigated in individuals three months or more after acute COVID-19 infection, and older than 18 years, including studies conducted in South American countries published between 2020 and 2022.

**Results** Six studies (four from Brazil and two from Ecuador) were analyzed. Regarding the type of study: three were cohorts, two were case reports, and one was cross-sectional. The main outcomes found were new pain (65.5%) and new chronic pain (19.6%), new headache (39.1%), daily chronic headache (13%), paresthesia (62%), in addition to neuropsychiatric diseases, such as generalized anxiety disorder (15.1%), post-traumatic stress syndrome (13.4%), depression and anxiety (13.5%), suicidal ideation (10.1%), and several cognitive disorders.

**Conclusion** Neurological and neuropsychiatric manifestations related to depression and anxiety, and cognition disorders are reported during the post-COVID-19 condition in South America. Symptoms associated with chronic pain appear to be associated with the condition. More studies on post-COVID-19 conditions are needed in the South America region.

## Resumo

**Antecedentes** A condição pós-COVID-19 é um grande desafio moderno na medicina e tem alto impacto global na saúde da população.

**Objetivo** Determinar as principais manifestações neurológicas e neuropsiquiátricas após a infecção aguda da COVID-19 nos países da América do Sul.

**Métodos** Trata-se de um estudo de revisão sistemática, registrado na plataforma PROSPERO seguindo o modelo PRISMA. Foram encontrados 4131 artigos com as estratégias de buscas empregadas. Investigaram-se manifestações neurológicas e neuropsiquiátricas em indivíduos com três meses ou mais desde a infecção aguda por COVID-19, maiores de 18 anos, incluindo estudos realizados em países da América do Sul publicados entre 2020 e 2022.

**Resultados** Foram analisados seis estudos (quatro do Brasil e dois do Equador). Em relação ao tipo de estudo: três eram coortes, dois relatos de casos e um transversal. Os principais desfechos encontrados foram em relação à dor nova (65,5%) e dor crônica nova (19,6%), cefaleia nova (39,1%), cefaleia crônica diária (13%), parestesia (62%), além de doenças neuropsiquiátricas como transtorno de ansiedade generalizada (15,1%), síndrome do estresse pós-traumático (13,4%), depressão e ansiedade (13,5%), ideação suicida (10,1%) e diversos distúrbios cognitivos.

**Conclusão** Manifestações neurológicas e neuropsiquiátricas relacionadas à depressão e ansiedade e distúrbios de cognição são relatados durante a condição pós-COVID-19 na América do Sul. Os sintomas associados a quadros de dor crônica parecem estar associados à condição. Mais estudos sobre condições pós COVID-19 são necessários na região da América do Sul.

## INTRODUCTION

The public health emergency caused by COVID-19 promoted the exhaustion of national health systems, evidenced the social determination on health, and generated an impact on global mortality. The number of deaths from COVID-19 exceeded 6.9 million deaths. 1 The Americas has the third highest number of COVID-19 cases, being the region with the highest number of deaths from the disease. South America had 629,005,065 cases and 2,917,071 deaths by the beginning of July 2023, and Brazil alone accounted for the second-highest number of deaths in the world. 1

After the World Health Organization (WHO) decree, on May 5, 2023, at the end of the health emergency of international interest, caused by SARS-CoV-2, 2 the current scenario in relation to the pandemic is considered favorable. However, the effects left on the health of the population, especially related to mental health, are undeniable. It is essential to recognize the extent of the impact of pandemic disease in physical, mental, and social health contexts, being topics of great interest within public health. 3

With the introduction of immunization and expansion of vaccination coverage, the number of cases and deaths from acute COVID-19 has reduced worldwide. However, since the second half of 2020, post-COVID symptoms have been noticed and studied by the scientific community. About 10 to 20% of the population that had COVID-19 may present symptoms and diseases for a period equal to or greater than 3 months - post acute phase of the disease, which cannot be explained for another reason. 4 This condition was initially termed Long COVID or Post-COVID Syndrome, and the WHO called it a post-COVID-19 condition. Although there is a high prevalence of symptoms after the acute phase of COVID-19 in several countries in the literature, ranging from 1.6-71% in the United Kingdom, 35-77% in Germany, and 49-76% in China, 5 to date, there is no consensus on the definition of the post-COVID-19 condition. 6 7 8

The post-COVID-19 condition is a major modern challenge in medicine, since there are no well-defined biochemical or radiological characteristics that help in the diagnosis, and there are several phenotypes and prognoses involved. 9 It is known that the effects of the post-COVID condition are multisystemic 10 and the main symptoms reported are persistent fatigue, shortness of breath, hair loss, and anosmia, in addition to the involvement of neuropsychiatric disorders, such as memory loss, brain fog, and depression, and can weaken millions of people worldwide, causing great harm to the health of the population. 9 10

The pathophysiology of post-COVID-19 conditions is still uncertain, 11 which, in turn, impacts the health of the world population leading to limitations of activities, decreased quality of life, and overload in health systems. Therefore, this study was carried out with the objective of determining the main neurological and neuropsychiatric manifestations following acute COVID-19 infection reported in the literature in South American countries.

## METHODS

This is a systematic review study, registered on the PROSPERO platform, under the protocol CRD42022337256, following the PRISMA model.

### Search strategy

The articles were searched in the Pubmed (MEDLINE), LILACS, SCOPUS, and Web of Science databases between May 2022 and June 2022, the last search being carried out on 25/07/2022. The descriptors used were: (post-acute COVID-19 syndrome OR long-COVID OR long haul COVID OR post-acute COVID syndrome OR persistent COVID-19 OR long hauler COVID OR long COVID OR post-acute sequelae and SARS-CoV-2 infection OR long haul COVID OR chronic COVID syndrome OR COVID-19/complications) AND (neurological disorder OR neuropsychiatric disorder OR neurology).

### Study selection

The selection of articles was carried out by two independent reviewers, according to the eligibility criteria. There was the removal of duplicates, selection of articles by reading titles, and then reading of abstract and full text, with the help of the Mendeley platform.

### Data extraction

Data were extracted by 2 independent reviewers and entered into Excel. The variables collected were: sociodemographic conditions, time since diagnosis of acute COVID-19, condition of acute COVID-19, neuropsychiatric manifestations described, and scales used to assess these conditions. In case of incomplete data, the corresponding author has been connected.

### Inclusion criteria

Cohort, case-control, cross-sectional, and case report studies were included. Neurological and neuropsychiatric manifestations were investigated in individuals aged 3 months or more since acute COVID-19 infection, older than 18 years. Studies conducted in South American countries published between 2020 and 2022, without the restriction on language of publication were included. Studies that did not present authors from South America were excluded when the place of performance was not mentioned in the text. The WHO definition of post-COVID-19 condition was used. 1

### Risk of bias

The risk of bias analysis was performed using the scale described by Looney (1998) used for cross-sectional studies that studied the prevalence of symptoms. Cohort studies were analyzed using the Newcastle-Ottawa scale. 13

## RESULTS

**Figure 1:**
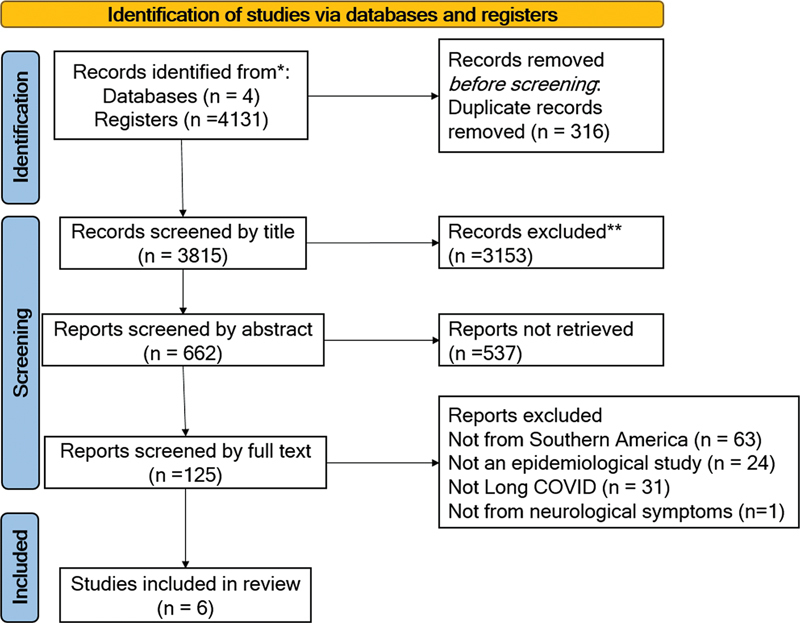
PRISMA flowchart of the study search process.

With the search strategies employed 4131 articles were found. After exclusions by duplicates and adopting inclusion criteria, 6 studies were analyzed ( Figure 1 ).

Among the included studies, 4 were from Brazil and 2 from Ecuador. Regarding the type of study, 3 were cohort, 2 were case reports and 1 was a cross-sectional study. As for the condition of acute COVID-19, 3 articles studied critical or hospitalized patients, 2 studies verified mild and moderate cases, without the need for hospitalization and 1 evaluated all types of acute manifestations, regardless of the type of manifestation of the case. The post-COVID-19 time described by the authors ranged from 3 to 18 months after the acute manifestations ( Table 1 ).

**Table 1:** Characteristics of the included studies

Author	Country	Study type	Time after acute COVID-19	Type of infection of acute COVID-19	n	
Damiano, 2022 14	Brazil	Cohort	3 months and 11 months	Hospitalized	701	
Del Brutto, 202 15	Ecuador	Cohort	6 and 18 months after onset of cases	Mild and moderate	78	50 with COVID-19
						28 without COVID-19
Soares, 2022 16	Brazil	Cross-sectional	4 months	Hospitalized	165	69 with COVID-19
						96 without COVID-19
Vasconcelos, 2022 17	Brazil	Case Report	8 months	Mild	1	
De Oliveira, 2021 18	Brazil	Case Report	3 months	Critic	1	
Del Brutto, 2022 15	Ecuador	Cohort	9 months after the start of the pandemic	All kinds	282	149 with COVID-19
						13 without COVID-19

In the study by Damiano et al. (2022) 14 using the Structure Psychiatric Interview: Clinical Interview Schedule Revised (CIS R) tool, the authors found a high prevalence of neuropsychiatric diseases 6 months after COVID-19. Common mental illnesses had a prevalence of 30% in the studied population. Among the diseases described are generalized anxiety disorder, depression, post-traumatic stress syndrome, obsessive-compulsive disorder, phobias, and mixed disorders (anxiety and depression). In addition, there were individuals with suicidal ideation and suicide attempts ( Table 2 ).

**Table 2:** Main outcomes found in the studies

Author	Outcome/parameter	Main findings	n (%)
Soares, 2022 16	Pain	New	30 (65.5)
		New chronicle	9 (19.6)
	Headache	New	19 (39.1)
		Daily chronicle	6 (13.0)
Del Brutto, 2021 19	Paresthesia		96 (62.0)
Damiano, 2022 14	Neuropsychiatric diseases	Generalized Anxiety Disorder (GAD)	106 (15.1)
		Post Traumatic Stress Syndrome	94 (13.4)
		Depression and Anxiety	95 (13.5)
		Suicidal ideation	71 (10.1)
			**Mean**
	Cognition	Mini-Mental Status Exam (MMSE)	8.27
		Trail Making Test (TMT-a)	65.5 seconds
		Digit Symbol Substitution Test (DSST)	13.11
		Boston naming test	5.2
		Memory complaint scale	15.57
		Verbal fluency	15.35
		Word listing	8.26
		Constructional praxis	8.26
		Word list recall	4.86
		Word list recognition	7.88
Del Brutto 2022 15	Cognition	Montreal Cognitive assesment (MOCA) prior	21.8
		Montreal Cognitive assesment (MOCA) 6 months post-COVID-19	19.7
		Montreal Cognitive assesment (MOCA) 18 months post-COVID-19	21.5

In another study, Soares et al. (2022) 16 analyzed pain according to the criteria of the International Association for the Study of Pain (IASP), 20 through interviews with participants. The symptom: new pain appeared in 65.2% of the population studied. Among them, 50% had a frequency of pain greater than 15 days a month. Pain chronicity was also present in 19.1% of individuals, with an average intensity of 6.7 on the Brief Pain Inventory (BPI) scale. 11 Headache was also reported by 39.1% of the individuals, the majority being reported with severe intensity 16 ( Table 2 ).

Del Brutto (2021), analyzed the strength using the Handgrip method. 19 Patients with COVID-19 infection had a higher prevalence of reduction in strength (62%) when compared to patients without previous infection (45%) of the disease. The drop in strength was also more pronounced for patients with previous COVID-19 infection (1.3) when compared to those without infection (1.0). The previous presence of COVID-19 predicted a higher risk of muscle strength reduction (OR= 2.27) 19 ( Table 2 ).

Del Brutto 2022 15 assessed cognition using the MOCA scale, 6 months and 18 months after COVID-19. The mean score was below the cutoff for the scale, 19.7 and 21.5, respectively. There was an improvement in the mean, but it did not return to the value before the infection, a mean of 21.8. 15

Damiano (2022) 14 used the scales, Memory Complaint Scale, Mini-Mental State Examination, verbal fluency, Boston naming test, word listing, Word List Recognition, Word List Recall, Trail Making Test *,* and Digit Symbol Substitution Test. The mean of the MMSE, DSST, and Boston recognition list scale showed cognitive alteration, while the TMT-a scale did not show the same 14 ( Table 2 ).

Damiano (2022) 14 analyzed cognitive alteration in patients with post-COVID-19 conditions, using the scales: Memory Complaint Scale (MCS), Mini-Mental State Examination (MMSE), Verbal Fluency, Boston Naming Test, Word Listing, Wordlist recognition, Wordlist recall and Digital Symbol Substitution Test (DSST). When the Trail Marking Test scale (TMT-a) was used, it was not possible to state the cognitive alteration ( Table 2 ).

Vasconcelos (2022), 17 in his case report, described the following symptoms: weight gain, excessive sleepiness, and cognitive alteration, with a final diagnosis of normal pressure hydrocephalus, after 8 months of COVID. 17 A demyelinating neuropathy, Lewis-Sumner syndrome, has also been described 3 months after COVID-19. 18

In the analysis of the risk of bias, the Newcastle Ottawa scale 13 was used in 2 studies, the first obtained a score in the selection of 2, in the comparison of 2, and in the outcome of 2. 15 The other study was obtained in selection 3, comparison 2, and outcome 2. 19 Two other studies were evaluated using the Loney scale (1998), which obtained 4/8 points 14 and 6/8 points. 16

## DISCUSSION

This study found a high frequency of neuropsychiatric manifestations, pain, and loss of muscle strength associated with the post-COVID-19 condition in South America. The data confirm the hypothesis that the population is suffering impacts on mental health throughout the COVID-19 pandemic, reinforcing the emerging problem that needs attention for the treatment of these conditions in the coming years.

Although Latin America is one of the regions with the highest number of cases and deaths in the world, 1 only 6 articles were found reporting neuropsychiatric manifestations of the post-COVID-19 condition throughout South America. The authors tried to expand the search to Latin America, and only 2 articles were added to the analysis, indicating that there is a need for more research throughout Latin America. A study that selected five Latin American countries to evaluate responses to the pandemic highlights the limitation of epidemiological data and highlights the deficiencies in disease monitoring in the selected countries. 21 When compared to the North American and European regions, Latin and South America do not have the same availability of data that allows an adequate population diagnosis. In addition to these problems, the South American region suffered from the lack of efficient regional governance, which aggravated the pandemic situation and compromised the economic recovery, and increased the repressed demand for services. 22 Therefore, the resumption of the offer of actions and services, added to the illness caused by post-COVID-19 conditions, are challenges that require health planning and surveillance in South America.

Two previous systematic reviews that addressed the post-COVID-19 condition found no studies in South American countries. 23 24 In the Brazilian scenario, only 4 articles addressed the topic, even though Brazil is the second country with the highest number of deaths. Justifying the scarcity of studies on the subject, it is suggested that the studies may not have happened very often, have been denied by journals, or not have a relevant neuropsychiatric manifestation in the population of South America, which would be contrary to the findings of the other regions. 25 The scarcity of studies could also indicate the underreporting of these conditions and the need for further scientific investigations, to enable adequate diagnoses and treatments.

The post-COVID-19 condition is characterized by the appearance of signs and symptoms after the acute phase of COVID-19. The exact time after acute infection that would characterize the post-COVID-19 condition still seems contradictory in the literature. The WHO definition establishes a period of development of new symptoms 3 months after initial SARS-CoV-2 infection, with these symptoms lasting at least 2 months with no other explanation. 7 The National Institute for Health and Care Excellence *(* NICE) in the United Kingdom established the period after 12 weeks. 6 The Centers for Disease Control and Prevention (CDC) understands any symptom manifested after four weeks of acute COVID-19 as a post-COVID-19 condition. 8 Thus, within the definition of the disease itself, there is a discrepancy between the periods analyzed, which can impact the methodological design and the results of studies on the subject.

The relationship between variations in the prevalence of psychiatric and neurological symptoms after acute COVID-19 is questionable, considering the lack of standardization of the periods of analysis of the post-COVID-19 condition. A study that analyzed the prevalence of neuropsychiatric symptoms from 3 to 6 months, and 6 to 9 months after acute COVID-19 observed that, when comparing the periods, the longest time (6 to 9 months) had a higher overall prevalence of symptoms such as depression, anxiety, and cognitive problems. 24 Another study showed that one month after COVID-19 infection, 54% of patients reported at least one symptom of a post-COVID-19 condition, this value being 56% after 2 to 6 months of the acute condition and 54% after 6 months or more. 26

Although there is a possible cut-off time for defining the onset of the post-COVID-19 condition (ranging from 4 to 12 weeks after acute infection), there is still no follow-up limit that defines how long the symptoms can be considered as a consequence of the infection and acute COVID-19. In the present systematic review, studies were included to observe the manifestations that developed in a period ranging from 3 to 18 months after the acute condition of the disease. Another systematic review that addressed neuropsychiatric manifestations selected data from patients evaluated for less than 12 weeks after acute COVID-19. 23 It is believed that the natural history of the post-COVID-19 condition is being studied, and studies with longer follow-up should be published in the medium and long term. These may be more enlightening about the causal link between acute COVID-19, time, and neuropsychiatric manifestations after COVID-19.

Most of the individuals with neuropsychiatric manifestations included in the analysis of this systematic review were cases that required hospitalization during the acute phase of COVID-19. However, the type of acute manifestation does not seem to influence the prevalence of neuropsychiatric manifestation and there are knowledge gaps that persist in this theme, requiring approaches that include this type of information in the analyses. 23

Regarding the influence of vaccination on post-COVID-19 conditions, a cohort conducted in Norway compared vaccinated and unvaccinated individuals, and found no differences between groups for symptoms such as dyspnea, fatigue, changes in smell/taste, concentration problems, evaluating from 3 to 15 months after the acute condition of COVID-19, except for memory problems, which were more reported among unvaccinated participants. 27 In the Netherlands, another cohort conducted to assess the effect of vaccination on recovery from symptoms of the condition post-COVID-19 found no therapeutic effects of vaccination against the symptoms of the disease over time. The authors state that there is an urgent need to understand the biological mechanism of the post-COVID-19 condition to enable preventive actions and provide treatment options. 28

When we analyze the mortality rates of the countries most affected by COVID-19 in South America, a study revealed that basic preventive measures, such as carrying out awareness programs, adherence to social distancing, wearing masks, providing adequate medical resources, together with increasing the vaccination rate, could effectively suppress the impact of the pandemic in the region. 29 However, as for the control of the pandemic, each country was influenced by the complex dynamics of internal policy and loco-regional inequalities to which they were inserted. 30 Brazil, being the largest country in South America, faced severe consequences of a national policy that did not adequately address the pandemic in a timely manner 31 and, with high contamination rates, in the medium to long term, a high prevalence of post-COVID conditions is expected. A Brazilian cohort identified that after 120 days of the acute phase of COVID-19, 80% of patients had persistent symptoms, the most prevalent being fatigue, dyspnea, cough, headache, and loss of muscle strength. 32

It is worth noting that, in the studies included in this review, there was a high frequency of pain, being defined as general pain in the body, which was not present before the COVID-19 infection. In the literature, body pain and headache represent symptoms of high prevalence in the post-COVID-19 condition, and have been causing great damage to the quality of life of individuals. 24 26 33 The symptoms of the condition after COVID-19 can affect several organs and systems, and fatigue (47%), dyspnea (32%), myalgia (25%), arthralgia (20%), and headache (18%) are the most prevalent. 34 Possible explanations for the development of pain suggest that post-COVID-19 conditions develop due to the persistence of inflammation in several organs. In the central nervous system, neuroinflammation occurs, causing the recruitment of microglia, activation of coagulation cascades, vasculopathy, oxidative stress, autoimmunity, and metabolic changes with neuronal dysfunctions. In the peripheral nervous system, the mechanism occurs through vasculopathies, oxidative stress, and autoimmune mechanisms, among other changes. 11

Several variables can help explain the causality of the development of neuropsychiatric diseases in post-COVID-19 conditions. First, there are physiological reasons for inflammatory origin that worsen in the body during COVID-19 infection. 35 Socioeconomic variables, such as instabilities at work, closure of non-essential sectors, lack of prospects for improving the health and economic situation, failures in government management to combat the pandemic, increased food insecurity in the population, financial difficulties, among other problems, may have contributed to the worsening of the mental condition of the population, regardless of the mode of contamination. 36 In addition, social isolation, of indisputable epidemiological importance in containing the spread of COVID-19 infection, can also result, in the long term, in damage to the mental health condition of the population. 37 In addition to contributing to the development of neuropsychiatric diseases, the pandemic and post-pandemic scenarios may aggravate existing disorders.

More studies are needed on the post-COVID-19 condition to clarify the population frequency in South America, adequate definition of symptoms and component diseases, and better understanding of times linked to the definition of what would be a post-COVID-19 condition and duration of its manifestations. The lack of a standard in cut-off time in relation to development and monitoring contributes to the time bias in relation to the theme. Some studies bring longer follow-up times, however, very long times will suffer from the tendency to present a greater number of disorders, including neuropsychiatric ones, which, not necessarily, will represent the post-COVID-19 condition.

This study has some limitations, one of which is due to the scarcity of studies on neurological and neuropsychiatric manifestations of the post-COVID-19 condition in South America. Another limitation is the absence of a study demonstrating the prevalence of general neuropsychiatric symptoms and not just specific symptoms. In addition, due to the limited number of articles found, it was not possible to perform the meta-analysis in this systematic review due to insufficient epidemiological measures on the studied event. However, the present study presents relevant results, emphasizes greater notoriety on the subject, and highlights the need for further studies on the post-COVID condition in South America, especially neuropsychiatric symptoms.

In conclusion, neurological and neuropsychiatric manifestations related to depression anxiety, and cognition disorders are reported during the post-COVID-19 condition in South America. Symptoms associated with chronic pain appear to be associated with the condition. More studies on post-COVID-19 conditions are needed in the South American region, for a better understanding of these manifestations and the correlation between them and the post-COVID-19 condition.

## References

[1] Coronavirus WHO. (COVID-19) Dashboard | WHO Coronavirus (COVID-19) Dashboard With Vaccination Data [Internet]. [citado 11 de julho de 2023]. Disponível em:https://covid19.who.int/

[2] OMS declara fim da Emergência de Saúde Pública de Importância Internacional referente à COVID-19 - OPAS/OMS | Organização Pan-Americana da Saúde [Internet]. [citado 11 de julho de 2023]. Disponível em:https://www.paho.org/pt/noticias/5-5-2023-oms-declara-fim-da-emergencia-saude-publica-importancia-internacional-referente

[3] Ashton J R Public mental health and the COVID-19 pandemic Ir J Psychol Med 1 4 10.1017/ipm.2021.16 PMC7985902 33583448

[4] A clinical case definition of post COVID-19 condition by a Delphi consensus, 6 October 2021 [Internet]. [citado 11 de julho de 2023]. Disponível em:https://www.who.int/publications-detail-redirect/WHO-2019-nCoV-Post_COVID-19_condition-Clinical_case_definition-2021.1

[5] Raman B Bluemke D A Lüscher T F Neubauer S Long COVID: post-acute sequelae of COVID-19 with a cardiovascular focus Eur Heart J 2022 43 11 1157 1172 35176758 10.1093/eurheartj/ehac031 PMC8903393

[6] Overview | COVID-19 rapid guideline: managing the long-term effects of COVID-19 | Guidance | NICE [Internet]. NICE; 2020 [citado 13 de julho de 2023]. Disponível em:https://www.nice.org.uk/guidance/ng188

[7] OMS emite definição clínica oficial da condição pós-COVID-19 | As Nações Unidas no Brasil [Internet]. [citado 13 de julho de 2023]. Disponível em:https://brasil.un.org/pt-br/150668-oms-emite-defini%C3%A7%C3%A3o-cl%C3%ADnica-oficial-da-condi%C3%A7%C3%A3o-p%C3%B3s-covid-19,https://brasil.un.org/pt-br/150668-oms-emite-defini%C3%A7%C3%A3o-cl%C3%ADnica-oficial-da-condi%C3%A7%C3%A3o-p%C3%B3s-covid-19

[8] Centers for Disease Control and Prevention [Internet]. 2022 [citado 13 de julho de 2023]. Post-COVID Conditions. Disponível em:https://www.cdc.gov/coronavirus/2019-ncov/long-term-effects/index.html

[9] The Lancet Understanding long COVID: a modern medical challenge Lancet 2021 398(10302):725 34454656 10.1016/S0140-6736(21)01900-0 PMC8389978

[10] RECOVER Mechanistic Pathways Task Force Mohandas S Jagannathan P Henrich T J Immune mechanisms underlying COVID-19 pathology and post-acute sequelae of SARS-CoV-2 infection (PASC) eLife 2023 12 e86014 37233729 10.7554/eLife.86014 PMC10219649

[11] Castanares-Zapatero D Chalon P Kohn L Pathophysiology and mechanism of long COVID: a comprehensive review Ann Med 2022 54 01 1473 1487 35594336 10.1080/07853890.2022.2076901 PMC9132392

[12] Loney P L Chambers L W Bennett K J Roberts J G Stratford P W Critical appraisal of the health research literature: prevalence or incidence of a health problem Chronic Dis Can 1998 19 04 170 176 10029513

[13] Stang A Critical evaluation of the Newcastle-Ottawa scale for the assessment of the quality of nonrandomized studies in meta-analyses Eur J Epidemiol 2010 25 09 603 605 20652370 10.1007/s10654-010-9491-z

[14] HCFMUSP COVID-19 study group Damiano R F Neto D B Oliveira J VR Association between chemosensory impairment with neuropsychiatric morbidity in post-acute COVID-19 syndrome: results from a multidisciplinary cohort study Eur Arch Psychiatry Clin Neurosci 2023 273 02 325 333 35633395 10.1007/s00406-022-01427-3 PMC9142732

[15] Del Brutto O H Rumbea D A Recalde B Y Mera R M Cognitive sequelae of long COVID may not be permanent: A prospective study Eur J Neurol 2022 29 04 1218 1221 34918425 10.1111/ene.15215

[16] “Pain in the Pandemic Initiative Collaborators” Soares F HC Kubota G T Fernandes A M Prevalence and characteristics of new-onset pain in COVID-19 survivours, a controlled study Eur J Pain 2021 25 06 1342 1354 33619793 10.1002/ejp.1755 PMC8013219

[17] Vasconcelos T MF Nóbrega P R Ferreira G M Normal pressure hydrocephalus associated with COVID-19 infection: a case report BMC Infect Dis 2022 22 01 216 35241017 10.1186/s12879-022-07184-x PMC8892823

[18] de Oliveira F AA de Oliveira Filho J RB Rocha-Filho P AS Multiple demyelinating sensory and motor mononeuropathy associated with COVID-19: a case report J Neurovirol 2021 27 06 966 967 34735692 10.1007/s13365-021-01024-5 PMC8567975

[19] Del Brutto O H Mera R M Pérez P Recalde B Y Costa A F Sedler M J Hand grip strength before and after SARS-CoV-2 infection in community-dwelling older adults J Am Geriatr Soc 2021 69 10 2722 2731 34124775 10.1111/jgs.17335 PMC8447376

[20] International Association for the Study of Pain (IASP) [Internet]. [citado 11 de julho de 2023]. International Association for the Study of Pain | IASP. Disponível em:https://www.iasp-pain.org/

[21] Benítez M A Velasco C Sequeira A R Henríquez J Menezes F M Paolucci F Responses to COVID-19 in five Latin American countries Health Policy Technol 2020 9 04 525 559 32874863 10.1016/j.hlpt.2020.08.014 PMC7451099

[22] Barros P S Gonçalves J de SB Samurio S EDesintegração econômica e fragmentação da governança regional na América do Sul em tempos de Covid-19.http://www.ipea.gov.br[Internet]. agosto de 2020 [citado 11 de julho de 2023]; Disponível em:https://repositorio.ipea.gov.br/handle/11058/10337

[23] Badenoch J B Rengasamy E R Watson C Persistent neuropsychiatric symptoms after COVID-19: a systematic review and meta-analysis Brain Commun 2021 4 01 fcab297 35169700 10.1093/braincomms/fcab297 PMC8833580

[24] Alkodaymi M S Omrani O A Fawzy N A Prevalence of post-acute COVID-19 syndrome symptoms at different follow-up periods: a systematic review and meta-analysis Clin Microbiol Infect 2022 28 05 657 666 35124265 10.1016/j.cmi.2022.01.014 PMC8812092

[25] Premraj L Kannapadi N V Briggs J Mid and long-term neurological and neuropsychiatric manifestations of post-COVID-19 syndrome: A meta-analysis J Neurol Sci 2022 434 120162 35121209 10.1016/j.jns.2022.120162 PMC8798975

[26] Short-term and Long-term Rates of Postacute Sequelae of SARS-CoV-2 Infection: A Systematic Review | Infectious Diseases | JAMA Network Open | JAMA Network [Internet]. [citado 11 de julho de 2023]. Disponível em:https://jamanetwork.com/journals/jamanetworkopen/fullarticle/2784918 10.1001/jamanetworkopen.2021.28568 PMC8515212 34643720

[27] Brunvoll S H Nygaard A B Fagerland M W Post-acute symptoms 3-15 months after COVID-19 among unvaccinated and vaccinated individuals with a breakthrough infection Int J Infect Dis 2023 126 10 13 36375693 10.1016/j.ijid.2022.11.009 PMC9651990

[28] RECoVERED Study Group Wynberg E Han A X Boyd A The effect of SARS-CoV-2 vaccination on post-acute sequelae of COVID-19 (PASC): A prospective cohort study Vaccine 2022 40 32 4424 4431 35725782 10.1016/j.vaccine.2022.05.090 PMC9170535

[29] Musa S S Tariq A Yuan L Haozhen W He D Infection fatality rate and infection attack rate of COVID-19 in South American countries Infect Dis Poverty 2022 11 01 40 35382879 10.1186/s40249-022-00961-5 PMC8983329

[30] Pablos-Méndez A Vega J Aranguren F P Tabish H Raviglione M C Covid-19 in Latin America BMJ 2020 370 m2939 32718938 10.1136/bmj.m2939

[31] Franceschini M CT Agrela de Andrade E Mendes R Akerman M Roque Andrade D Madalena de Campos Lico F Information, control and health promotion in the Brazilian context of the pandemic Health Promot Int 2022 37 01 daab032 34114018 10.1093/heapro/daab032 PMC8394828

[32] Bonifácio L P Csizmar V NF Barbosa-Júnior F Long-Term Symptoms among COVID-19 Survivors in Prospective Cohort Study, Brazil Emerg Infect Dis 2022 28 03 730 733 35133956 10.3201/eid2803.212020 PMC8888217

[33] Rogers J P Watson C J Badenoch J Neurology and neuropsychiatry of COVID-19: a systematic review and meta-analysis of the early literature reveals frequent CNS manifestations and key emerging narratives J Neurol Neurosurg Psychiatry 2021 92 09 932 941 34083395 10.1136/jnnp-2021-326405

[34] TLC Study Group Aiyegbusi O L Hughes S E Turner G Symptoms, complications and management of long COVID: a review J R Soc Med 2021 114 09 428 442 34265229 10.1177/01410768211032850 PMC8450986

[35] Hu B Guo H Zhou P Shi Z L Characteristics of SARS-CoV-2 and COVID-19 Nat Rev Microbiol 2021 19 03 141 154 33024307 10.1038/s41579-020-00459-7 PMC7537588

[36] Jaspal R Breakwell G M Socio-economic inequalities in social network, loneliness and mental health during the COVID-19 pandemic Int J Soc Psychiatry 2022 68 01 155 165 33287610 10.1177/0020764020976694 PMC8793303

[37] Pai N Vella S L The physical and mental health consequences of social isolation and loneliness in the context of COVID-19 Curr Opin Psychiatry 2022 35 05 305 310 35787541 10.1097/YCO.0000000000000806

